# Hypertension Predisposition and Thermoregulation Delays in Adolescents with Polycystic Ovary Syndrome: A Pilot Study

**DOI:** 10.3390/children9030316

**Published:** 2022-02-25

**Authors:** Styliani Geronikolou, Flora Bacopoulou, Stavros Chryssanthopoulos, Dennis V. Cokkinos, George P. Chrousos

**Affiliations:** 1 Clinical, Translational and Experimental Surgery Research Centre, Biomedical Research Foundation Academy of Athens, Soranou Ephessiou Str. 4, 11527 Athens, Greece; fbacopoulou@med.uoa.gr (F.B.); s.chryssanthopoulos@gmail.com (S.C.); dcokkinos@bioacademy.gr (D.V.C.); chrousos@gmail.com (G.P.C.); 2 University Research Institute of Maternal and Child Health & Precision Medicine, School of Medicine, National and Kapodistrian University of Athens, “Aghia Sophia” Children’s Hospital, Levadias Street 8, 11527 Athens, Greece; 3 Center for Adolescent Medicine, UNESCO Chair in Adolescent Health Care, First Department of Pediatrics, School of Medicine, National and Kapodistrian University of Athens, “Aghia Sophia” Children’s Hospital, Thivon Street 1, 11527 Athens, Greece

**Keywords:** polycystic ovary syndrome, thermoregulation, arterial stiffness, arterial thickness, bioimpedance, hypertension, heart rate variability, PCOS

## Abstract

Background: Polycystic ovary syndrome (PCOS) is a heterogeneous disorder in which clinical, sonographic, and endophenotypic features have been underinvestigated or inconclusive, especially in the early stages of the disease (adolescence/young adulthood). Objective: This prospective pilot study focused on the differences of multiple physiological functions between Greek adolescent/young adult females suffering from PCOS and age- and body mass index (BMI)- matched healthy controls. Study design: Nineteen PCOS patients and eighteen healthy controls (aged 13 to 23 years) were studied for: (i) biochemical and hormonal dysfunction by measuring circulating glucose, insulin, and androgen levels; (ii) arterial stiffness with pulse wave analysis (PWA) by Sphygmocord; (iii) intima-media thickness (IMT) by ultrasound; (iv) heart rate variability (HRV) by Task Force Monitor; and (v) QT, QRS, QT, P, QRSD by electrocardiogram (ECG). Statistical analysis included Hedge’s g correction for small samples bias, and the results are shown using the Hedge’s g effect size and 95% CI, in line with precision medicine prerequisites. Results: Significant differences in pulse wave velocity (PWV) (g = 0.964 [0.296, 1.632]), subendocardial viability ratio (SEVR) carotid (g = −0.679 [−1.329, −0.030]), pulse pressure (PP) carotid (g = 0.942 [0.275, 1.608]), systolic pressure (SP) carotid (g = 0.785 [0.129, 1.440]), free-testosterone (g = 0.677 [0.042, 0.312]), and Delta4-androstenedione (g = 0.735 [0.097, 0.373]) were observed between PCOS patients and controls. No differences were detected in the remaining endocrine and PWA or ECG biomarkers. Conclusions: Our multidisciplinary approach showed early onset of vascular dysfunction, predisposition to hypertension, thermoregulation delays, and metabolic syndrome changes in adolescent/young adult PCOS.

## 1. Introduction

Polycystic ovary syndrome (PCOS) is the most common endocrine disorder in women of reproductive age, with a prevalence of up to 15%, depending on the ethnicity of the subjects and the diagnostic criteria employed [1].

PCOS is a heterogeneous disorder of both genetic and epigenetic etiology [2,3]. Obesity, and especially excess abdominal adipose tissue, aggravate the endocrine and metabolic dysregulation of the syndrome [3]. Women with PCOS are at increased risk of insulin resistance, metabolic syndrome manifestations, and ultimately, type II diabetes mellitus [1,4]. In addition, the syndrome has adverse cardiovascular implications [5], clinical manifestations of systemic “para-inflammation” and disorderly sleep [6], hypertension [7,8], dermatological manifestations [9], and anxiety and/or depression [10].

Carotid artery intima-media thickness (IMT), an established marker of future cardiovascular disease, which strongly and linearly correlates with age, has been examined in adult women with PCOS [11]. Of note, IMT has been associated with childhood and adult obesity, and other chronic diseases (e.g., depression, metabolic syndrome, hypercholesterolemia, hypertension, chronic kidney disease, obstructive sleep apnea, cognitive impairment, as well as polycystic ovaries syndrome [12,13,14,15,16,17].

Although the clinical, biochemical, hormonal, and sonographic symptoms and signs of PCOS may manifest in adolescence [18,19,20], data on the autonomic and cardiovascular function of adolescent or young adult women with PCOS are limited [21,22,23,24,25,26].

The complexity of PCOS makes a multi-system research approach essential. To this end, our study examined potential differences in systemic endophenotypic features between Greek adolescent/young adult females with and without PCOS. Associations of the subjects’ biochemical/hormonal, cardiovascular, and autonomic biomarkers with their body composition parameters were assessed.

## 2. Population and Methods

### 2.1. Population

Study participants included Greek adolescent and young adult females, aged 13 to 23 years, with PCOS, as well as body mass index (BMI)- matched healthy controls. They all presented to the Centre of Adolescent Medicine and UNESCO Chair in Adolescent Health Care of the First Department of Paediatrics, at the “Aghia Sophia” Children’s Hospital, in Athens, Greece, over a period of one year, from June 2013 to November 2014. They all underwent a detailed physical, biochemical, hormonal, and sonographic assessment. Polycystic ovary syndrome was diagnosed if at least 2 years had elapsed from menarche, on the basis of at least two of the three Rotterdam criteria, i.e., (a) oligo -anovulation, (b) clinical and/or biochemical hyperandrogenism, and (c) sonographic appearance of polycystic ovaries. Study participants were allocated to 2 groups, the PCOS group (mean age 16.8 ± 3.3 years) and the healthy control group (mean age 16.9 ± 2.1 years). Exclusion criteria included pregnancy, another genetic or endocrine disorder, psychiatric illness, chronic use of medications, or uptake of hormonal contraceptives.

All measurements, with the exception of the hormonal assays, were performed at the Cardiac and Vascular Laboratory of the Clinical, Translational, and Experimental Surgery Research Centre of the Biomedical Research Foundation of the Academy of Athens. The study adhered to the Helsinki Guidelines of Good Clinical Practice [27] and was approved by the Biomedical Research Foundation of the Academy of Athens Institutional Review Board (code number 121/2013-4-26). Written informed consent was obtained from all participants and the parents of those who were younger than 18 years, at the enrolment into the study.

### 2.2. Measurements

**Figure 1:**
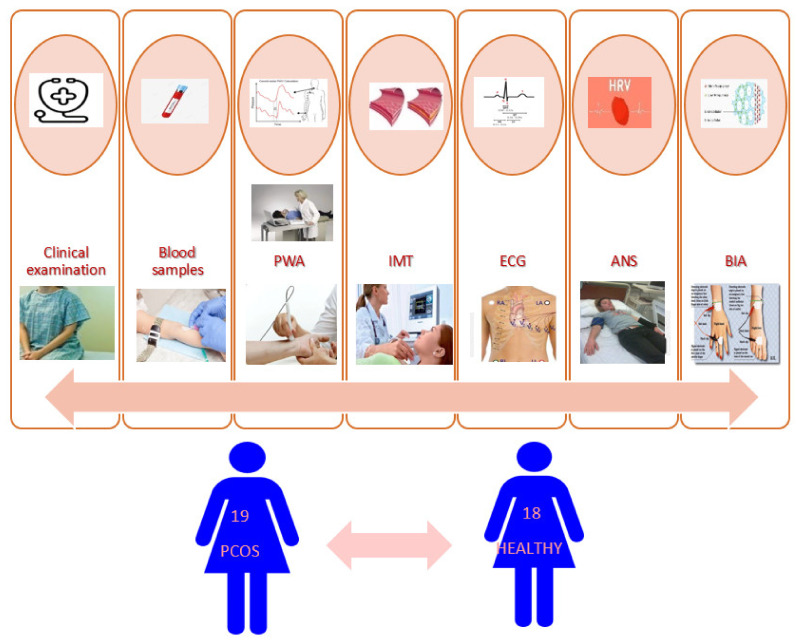
Study design.

The study design and the procedures taken are illustrated in Figure 1.

Blood samples: Twenty minutes after venipuncture and blood collection in EDTA tubes, the samples were centrifuged at 2000× *g*, at 4 °C, for 20 min. The plasma aliquots were then stored at −80 °C in the Biomedical Research Foundation of the Academy of Athens until analysis at the Endocrine Laboratory of the First Department of Pediatrics of the National and Kapodistrian University of Athens. Serum concentrations of insulin, free-testosterone (free-T), Delta4-androstenedione (D4-andro), and dehydroepiandrosterone sulphate (DHEAS) were assayed by an Immulite 2000 analyzer (Siemens Healthcare Diagnostics Products Ltd., Camberley, UK), with two-site chemiluminescent immunometric assays. Glucose was assessed with the photometric method. Insulin resistance was calculated by the homeostasis model assessment-insulin resistance index according to the initial formula [HOMA-IR = insulin (expressed in mU/L) × glucose (expressed in mg/dL)/405].

The electrocardiographic recording (ECG) was performed with a Philips Pagewriter 200. For practical purposes, Bazett’s formula (measured QT interval divided by the square root of the R-R interval) remains the most commonly used method for determining the rate corrected QT interval (QTc). Normal rates are between 300–450 ms.

Cardiac function was also assessed by measuring autonomic nervous system (ANS) electrophysiologic markers. Heart rate variability (HRV), a valid tool assessing autonomic tone (7.15), was recorded in each subject with the Holter ECG. Each HRV component determined was linked to each of the limps of the ANS; thus, the root mean square of the successive differences (RMSSD) and high frequencies (HF) were linked to the parasympathetic tone, while very low frequencies (VLF), and low frequencies (LF), the standard deviation of the N-N interval (SDNN), the standard deviation of the average N-N interval (SDANN), the mean of the standard deviation in all 5-min segments of a 24-h recording (ASDNN), the number of pairs of NN interval that are longer than 50 msec (NN50), and the proportion of NN50 divided by the total number of NNs (pNN50), were all linked to the mixed autonomic activity (Task Force of the European Society of Cardiology and the North American Society of Electrophysiology, 1996).

Furthermore, VLF reflects the underlying periodicities in the heart rate at frequencies of q 25 s to q 5 min (0.0033–0.04 Hz). The time-domain indices of HRV were assessed by SsER Light Extend Compact Holter Recorders-GE Healthcare (General Electric Med systems Information Technologies Inc., Tokyo, Japan).

Arterial stiffness was measured in the sitting position, using pulse wave analysis (PWA) by a SphygmoCor device. SphygmoCor AtCor Medical, Sydney, NSW, Australia, was used to transform the radial and carotid pressure waveform into an aortic pressure one by using a validated transfer function. Three successive measurements were recorded. Augmented pressure was calculated as the difference between the second and the first systolic peak and the augmentation index was calculated as the ratio between the augmented pressure, pulse pressure (PP), and subendocardial viability ratio (SEVR), a pressure–time integral ratio derived from pressures measured in the aorta and LV, which represents myocardial oxygen supply and demand. In this study, SEVR was calculated as per the manufacturer: it was approximated automatically using the following equation: SEVR = diastolic aortic area/systolic aortic area [28]. It is worth noting that the 60 mmHg PP value has been chosen as the cut point of epidemiological data corresponding to a significant PP-induced increase in cardiovascular risk [29,30].

Vascular function was also assessed by measuring the intima-media thickness (IMT) biomarker, i.e., the combined thickness of the arterial intima and medial layers in the carotid arteries of each study participant. Intima-media thickness was measured by B mode ultrasonography (GE Medical Systems Vivid 7 Pro Transducer 12L), with the subjects lying supine.

Study participants, free of metallic dental braces or metallic orthopedic implant, underwent body composition analysis by bioelectrical impedance, using a BIA phase-sensitive system (single-frequency 50 kHz BIA 101 RJL, Akern Bioresearch, Florence, Italy). Subjects were assessed in the supine position, with their limbs slightly spread apart from the body, shoes and socks removed, and contact areas scrubbed with alcohol immediately before electrode placement. Electrodes (BIATRODES Akern Srl; Florence, Italy) were placed proximally to the phalangeal–metacarpal joint on the dorsal surface of the right hand and distal to the transverse arch on the superior surface of the right foot. Sensor electrodes were placed at the midpoint between the distal prominence of the radius and ulna of the right wrist, and between the medial and lateral malleoli of the right ankle in each study participant.

Bioelectrical impedance phase angle (PhA°) detects changes in tissue electrical properties and reflects the relative contributions of fluid and cellular membranes of the human body [31]. Phase angle differs significantly between health and disease states, with lower values suggesting cell death or decreased cell integrity and higher values suggesting more intact cell membranes [32]. In this study, PhA was derived from conditions under 50 kHz according to the following Equation (1):

PhA (°) = arctangent Xc/R ((Xc/R) × (180/*π*) (1)

where Xc: reactance; R: resistance, and *π*: 3.14.

Basal metabolic rate (BMR), which is the amount of energy expended while at rest in a neutrally temperate environment, in the post-absorptive state (meaning that the digestive system is inactive, which requires about twelve hours of fasting) was calculated by indirect calorimetry. Abdominal adipose tissue (AAT) in cm^2^ and extra-cellular water to body cell mass ratio (ECW/BCM) measurements were predicted by the equations suggested by Bray and Ramirez and validated by the studies of Tsigos et al. and Wan et al. [33,34,35,36].

All measurements were performed after an overnight fast, between 8:30 and 10:30 am. Study participants had been asked to refrain from coffee, tobacco, or alcohol consumption or any vigorous exercise for 24 h before testing (as suggested by the European Society of Parenteral and Enteral Nutrition (ESPEN) [37], the European Society of Cardiology, and the North American Society of Electrophysiology guidelines [38].

### 2.3. Statistical Analysis

**Figure 2:**
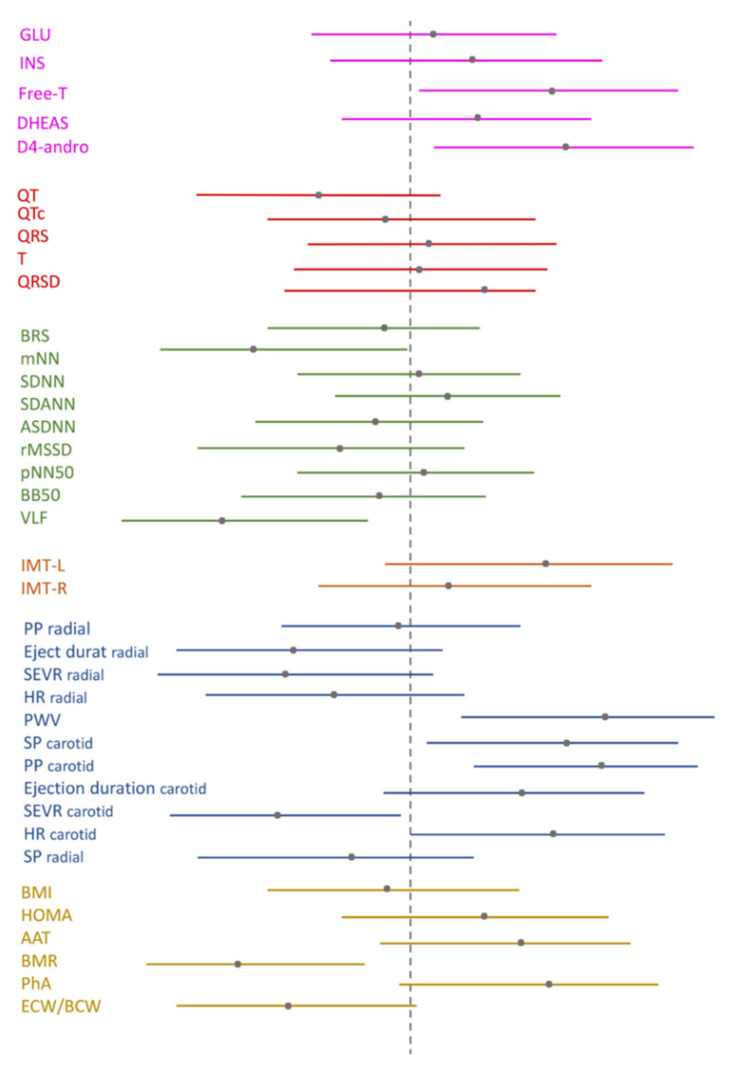
Differences between case-control groups are presented in Hedge’s g (correction of Cohen d for small samples) and 95% Confidence Intervals.

Group descriptives were evaluated in SPSS21. To overcome the variance implications due to the relatively small sample size or the possible disease particularities, the differences between PCOS and healthy subjects were estimated and presented in effect sizes. The preferred effect size was the modification proposed by Lipsey and Wilson (2001) and Durlak (2009) on Hedge’s g statistics formula (Hedges’ g is a variation of Cohen’s d that corrects the bias due to small sample sizes) [39,40]. It can be interpreted in the same way as Cohen’s d, whereby 0.2 represents a small effect, 0.5 a medium effect, and 0.8, a large effect [41]. The evaluations were performed in R software. The calculated effect sizes, standard error, variance, and 95% CI are illustrated in Figure 2, per each function: biochemical/endocrine (Figure 2), cardiac (Figure 2), arterial stiffness (Figure 2), body composition, and metabolism (Figure 2). Different forest plot colors for the different functions were chosen in Figure 2.

Correlation coefficients (r) are converted to Hedge’s g via the following formula: g = $\frac{2r}{\sqrt{1-r2}}$, where r is the Spearman coefficient. The resulting significant coefficients are converted to Hedge’s g and presented in Table 1.

The non-parametrical Spearman test was used for estimating the correlations for age, available in Supplementary Materials.

Finally, confidence intervals are calculated as follows: CI = $\bar{x}$ ± z* σ/√n, where CI: confidence interval, $\bar{x}$: mean, z: z confidence level (here, 0.95), n: sample size, σ: standard deviation.

## 3. Results

Nineteen PCOS patients (age range 13–24 years, BMI range 19.5–32.1 kg/m^2^) and eighteen healthy controls (age range 13–22 years, mean, BMI range 21.5–35.7 kg/m^2^), participated in the study. All participants’ characteristics in means and standard deviation are included in Table S1. 

Biochemical/hormonal profile: Significant differences in free-T (g = 0.677 [0.042, 0.312]), and *D4-dione* (g = 0.735 [0.097, 0.373]) levels between PCOS patients and controls were observed (Figure 2).

Electrocardiographic profile: No differences in ECG measurements between the PCOS and healthy groups were observed (Figure 2).

Autonomic profile: All measurements are presented in Table 1 and Figure 2. Significant differences were found in VLF and power spectral density RR between the two groups; however, no significant differences were observed in any of the time domain markers of HRV (Figure 2).

Vascular profile: No differences in IMT measurements in either carotid artery between the PCOS and healthy groups were observed (Figure 2). Significant differences in PWV (g = 0.964 95% CI [0.296, 1.632]), SEVR carotid (g = −0.679 [−1.329, −0.030]), PP carotid (g = 0.942, 95% CI [0.275, 1.608]), and SP carotid (g = 0.785, 95% CI [0.129, 1.440]) biomarkers (Table 1, Figure 2).

Body composition and metabolism: The body composition parameter effect sizes of the study participants of both groups are presented in Table 1 and Figure 2. No differences were found in any of the body composition or metabolic measures between the two groups.

A statistically significant strong correlation was observed only between BMR and PhA, Hedge’s g= −0.829, 95% CI [−1.543, −0.115] only in the PCOS group (Table 2). The same was calculated between PP and SP: Hedge’s g = −1.158, 95% CI [−2.252, −0.063] (Table 2).

Age was not correlated with any of the significantly altered characteristics (that are described in Supplementary Materials Table S2).

## 4. Discussion

### 4.1. Principal Findings

PCOS patients manifested slightly decreased levels in the circulating levels of free-T and D4-andro. Both groups studied here showed normal electrocardiograms and no significant arrhythmias, however, the QT was shorter in PCOS than in their counterparts (Figure 2). No significant alterations of other electrocardiographic measurements were observed between the two groups.

Our study revealed a significant decrease in the VLF component of HRV in adolescent and young adult women with PCOS compared to their healthy controls. In our population, however, no significant correlation was found between IMT and the area of abdominal adipose tissue assessed by bioimpedance. Additionally, altered arterial stiffness markers were observed in our PCOS compared to the healthy group. While we found no differences in BMI, in abdominal adipose tissue, or other bioimpedance parameters, including BMR, PhA, and ECM/BCM between the two groups, a strong correlation between BMR and phase angle was observed in the PCOS group only.

### 4.2. Comment

The heterogeneity of PCOS physiology points to the interaction of genetic and environment-related factors, neuroendocrine changes, and endocrine/metabolic changes like insulin resistance, hyperinsulinemia, altered anti-Müllerian hormone and adiponectin levels, and total and abdominal adiposity. Acne, hirsutism, hyperandrogenemia, and chronic oligo-anovulation usually precedes ovarian morphology assessment in adolescents with PCOS. This process was followed here: our PCOS patients came to the clinic with the above physical complaints. Free-T levels suppression were then determined for corroboration [42,43]. Circulating free-T and D4-andro concentrations were not elevated, as hyperandrogenemia is not a sine qua non for PCOS diagnosis, according to the Rotterdam criteria. Besides, the sample size is small and the age group very young. A second verification in the same pathway was revealed when we compared the effect sizes, with the effect of D4-andro found greater than that of free-T g_Δ4_ > g_T_ (Figure 2). Moreover, the androgens decrease may be influenced by socio-economic, environmental, and life circumstances, a diminished sense of well-being; dysphoric mood and/or blunted motivation, follicular phase sampling [44].

Normally, the ECG in childhood and adolescence differs from that of adults [45]. Thus, the child and adolescent T wave pattern, particularly in precordial leads, progressively changes from birth to early adult life. Although the rate at which this change occurs varies considerably from one individual to another, some generalizations can be made [45]. Additionally, detecting prolongation of the QT interval is important in the identification of individuals at risk of arrhythmia. The early-stage and young age of our target group, was probably the reason for the absence of abnormalities in the ECG.

The activities of the sympathetic and parasympathetic limps of the ANS of the heart are valid biomarkers of cardiac function [38,46,47]. VLF has been considered a biomarker of neurohormonal fluctuations, while limited data report that VLF can be modulated by joint action of the renin-angiotensin-aldosterone system (granted that it is reduced by angiotensin-converting enzyme inhibition) and the parasympathetic tone (as it can be blocked by atropine), while it is not affected by the sympathetic nervous system (granted that it is not influenced by the beta-adrenergic blockade) [34,43]. This finding needs to be further investigated in larger cohorts, so as to find its underlying contributors. VLF is implicated in thermoregulation [48,49].

The absence of significant differences in the time domain indices of heart rate variability between the two groups indicates no predisposition to cardiac disease related to these indices, at least at this early stage of the disease.

Increased IMT is associated with male gender, high circulating cholesterol, and hypertension [46,50]. It is a biomarker of arterial wall thickness and a strong predictor of cardiovascular disease in males aged < 60 years [51]. In women with PCOS, endothelial structure and function have been incompletely evaluated [51,52]. We studied arterial thickness expressed as IMT and found no significant differences between the two groups. Our observations are in agreement with previous findings in adolescents with PCOS [53,54,55,56]. High IMT values in PCOS were reported in women in early adulthood (associated with decreased sex hormone-binding globulin) [57] and adulthood [58,59,60,61,62,63,64,65] in obese women [50,66,67] and patients with juvenile rheumatoid arthritis [68]. Our study participants were adolescent and young adult women with PCOS and proper controls matched for BMI, so as to overcome the strong confounding associations of age and BMI with arterial thickness in PCOS [68]. Increased IMT has been positively related to abdominal adiposity in adult women with PCOS [68]. Our null result in abdominal tissue and the arterial thickness could be due to the younger ages of our subjects or to the different methods employed.

The two groups, patients and controls were age- and BMI-matched so as to overcome the strong confounding positive association of age and BMI with arterial thickness and stiffness in PCOS [68]. PWV, PP, and IMT are strong predictors of cardiovascular disease in males aged < 60 years according to meta-analyses [51,68,69]. Our study had a completely different target population, but these variables were included and measured. Notably, endothelial structure and function have been incompletely evaluated in PCOS [65]. Regarding arterial stiffness, the close agreement between sitting and supine measurements in the Nurnberger et al. study suggested that arterial stiffness indices can be reliably measured in the sitting position [70].

Reduced SEVR, measured in the carotid artery, has been associated with metabolic syndrome, independently of glucose tolerance [71] and short-duration type 2 diabetes mellitus in women [72]. The observed significantly decreased carotid SEVR in our PCOS compared to the healthy group provides evidence that the characteristic cardiometabolic implications have their onset already in adolescence, even if the cardiovascular consequences appear later in life.

PP is the difference between the systolic and diastolic blood pressure readings. It represents the force that the heart generates each time it contracts. Local PP measure at the site of the arterial segment whose geometry is studied (due to the physiological PP amplification between central and peripheral arteries), has been recommended, so as to determine the relationship between cyclic stress and arterial remodeling [73]. This relation was confirmed in our PCOS subjects, where the effect size of the correlation was g = −1.158, 95% CI [−2.252, −0.063] for the left carotid (elastic) artery. This evidence enhances the above finding that our population’s physiological features point to a tendency to hypertension. The radial PP (muscle artery) showed no significant differences, however, this is of small value, as the radial PP is not a cardiovascular disease predictor- [73]. Increased stiffness of the aorta and large arteries leads to an increase in PP through a reduction in arterial compliance reflected in cyclical wave changes.

Data regarding body composition assessed by BIA in PCOS vary from increased body fat [74,75], to no differences in body composition compared to healthy women [76]. Three studies [77,78,79] that used MRI to assess body fat distribution in lean, normal weight, and obese adults, failed to report preferential visceral fat accumulation in PCOS cases vs. controls. We observed no differences in BMI, abdominal adipose tissue, or other bioimpedance parameters, including BMR, PhA, and ECM/BCM between the PCOS and healthy groups, a finding that may be explained by our design (BMI match between the comparison groups). AAT is a marker of metabolic syndrome [80], severe obesity, atherogenesis, etc. [81]. BMR is the amount of energy expressed in calories that a person needs to keep her body functioning at rest. The absence of differences in these biomarkers suggests no obvious metabolic differences between the two groups. ECM/BCM has been associated with malnutrition [82] morbidity, and/or mortality [83]. As the sensitive malnutrition marker, ECM/BCM was similar in the study groups, we concluded that our study was free of nutrition-related confounding. Yet, we found a positive correlation between BMR and PhA—a finding that has been previously observed in anorexia nervosa [84] and severe obesity [85].

PhA is a bioimpedance marker of inflammation, and PCOS has been considered an inflammatory state, attributed to obesity or AAT excess. Yet, as our two groups had the same BMI and we observed no difference in AAT, the correlation of BMR with PhA found could be PCOS-specific. Relatively activated circulating mononuclear cells in PCOS might be related to hyperandrogenemia rather than AAT or insulin resistance [86].

The syndrome’s reproductive and metabolic implications suggest that women with PCOS are at serious risk of infertility, insulin resistance, metabolic syndrome, and type II diabetes mellitus [1,4].

Power spectral density was decreased in our PCOS subjects, which together with the VLF changes observed point toward thermoregulatory delays in these subjects [49]. Thermoregulation delays have been observed in adult obese PCOS patients, as well as in adult transgender men [87]. The phenomenon has been attributed to hyperandrogenemia, an established vasculature moderator in females [88].

Finally, by comparing the side effects of the differences observed, we determined that the highest effect was that of PWV; this observation points to premature endothelial calcification in peripheral vessels which, apparently, precedes the cardiovascular changes associated with the syndrome. Calcification is a complex, long-lasting process associated with cardiometabolic abnormalities and lifestyle [89]. Interestingly, our study suggests that early underlying metabolic changes contribute to future atherosclerosis and hypertension in PCOS, even independently of BMI.

Such cost-effective, as well as time-saving and clinician-friendly methodology (ANS, body composition, inflammatory indices), prove to be beneficial to diagnosticians as they suggest early prevention measures.

Our study provides evidence on ethnic-related PCOS morbidity. We performed a multisystem investigation, which attempted to contribute to the resolution of the long-standing conflict regarding the diagnostic criteria of the syndrome.

A previous in silico study of ours revealed genes/gene products involved in the PCOS interactome: leptin, interleukin-1, interleukin-6, vasopressin, tumor necrosis factor-alpha, etc., interacted with androgens [90]. Some of these factors may explain the thermoregulatory abnormalities we observed in adolescence, suggesting that they may exist from the early stages of the disease, irrespectively of BMI.

Further research should be done on the thermoregulatory, metabolic, and ANS changes in PCOS, as they emerge in adolescence and young adulthood, using evolving diagnostic tools and new basic science knowledge. For example, future work would examine nitrates/nitrites or citrulline, nitric oxide synthase metabolites, adiponectin, resistin, or leptin concentrations so as to confirm the herein revealed endothelial dysfunction, especially if correlated to hormones levels.

The non-invasive method of advanced bioimpedance was used in this study successfully in assessing differences in body composition, metabolism, and inflammation between patients and controls.

Studying a clinical problem in its early stages in a comprehensive manner is a strength. PCOS is considered a gynecological problem, as well as a multisystem disorder. To this end, our multisystem adolescent/young adulthood-targeted study attempted to unravel the early stages of the syndrome’s pathogenetic mechanisms. The study benefited from a new non-invasive but established technology (bioelectrical impedance or bioimpedance).

The small subject population studied was a limitation of the study. We opted to evaluate effect sizes so as to reduce the variance. The Hedge’s g statistics formula was adopted as it is a variation of Cohen’s d that corrects the bias due to small sample sizes [39,40,91].

## 5. Conclusions

Although there were differences in arterial wall thickness, most electrocardiographic features, ANS tone, and amount of abdominal adipose tissue were not significant between the adolescent/young adult women with PCOS and the age- and BMI-matched healthy controls, however, significant differences were found in arterial wall stiffness, BMR, and VLF. The latter marker is related to the activity of the renin-angiotensin-aldosterone axis, suggesting the early onset of hormonal changes and/or cardiometabolic and thermoregulatory manifestations related to the syndrome. These observations, together with the strong correlation between BMR and the inflammatory marker PhA in the PCOS subjects, suggest that starting at this young age, PCOS patients should be closely monitored and urged to follow a healthy lifestyle [83].

## Figures and Tables

**Table 1:** Differences between case-control groups are presented in Hedge’s g (correction of Cohen d for small samples) and 95% Confidence Intervals, Standard Error, and Variance. Significant variables are marked in bold characters.

Function	Marker	Hedges’s g	Standard Error	Variance	Lower Limit	Upper Limit
Metabolism	BMI (kg/m^2)^	−0.103	0.322	0.104	−0.735	0.528
	HOMA	0.313	0.317	0.100	−0.308	0.933
	AAT (cm^2^)	0.510	0.327	0.107	−0.131	1.151
	**BMR** (kcal/day)	−0.890	0.338	0.114	−1.553	−0.228
	PhA (°, degrees)	0.613	0.330	0.109	−0.033	1.259
	ECW/BCM	−0.626	0.330	0.109	−1.272	0.021
Biochemical/Endocrine	Glucose	0.115	0.315	0.099	−0.502	0.732
	Insulin	0.237	0.316	0.100	−0.382	0.855
	Free-T	0.677	0.324	0.105	0.042	1.312
	DHEAS	0.192	0.315	0.099	−0.426	0.810
	D4-andro	0.735	0.325	0.106	0.097	1.373
Heart	QT	−0.480	0.327	0.107	−1.120	0.160
	QTc	−0.075	0.322	0.104	−0.706	0.556
	QRS	0.063	0.322	0.104	−0.568	0.694
	T	0.052	0.322	0.104	−0.579	0.683
	QRSD	0.040	0.322	0.104	−0.590	0.671
Autonomic Nervous System	BRS (ms/mmHg)	−0.155	0.322	0.104	−0.786	0.477
	mNN	0.647	0.331	0.109	−1.295	0.000
	SDNN	0.063	0.322	0.104	−0.568	0.693
	SDANN	0.231	0.323	0.104	−0.402	0.864
	ASDNN	−0.217	0.323	0.104	−0.849	0.416
	rMSSD	−0.390	0.325	0.106	−1.027	0.247
	pNN50	0.044	0.322	0.104	−0.587	0.675
	BB50	−0.210	0.323	0.104	−0.842	0.423
	**VLF**	−0.905	0.339	0.115	−1.569	−0.242
Arterial Thickness & stiffness	IMT-L	0.593	0.329	0.106	−0.052	1.232
	IMT-R	0.142	0.322	0.104	−0.490	0.773
	PP radial	−0.048	0.322	0.104	−0.679	0.583
	Eject duration radial	−0.552	0.329	0.106	−1.195	0.091
	SEVR radial	−0.565	0.328	0.108	−1.209	0.071
	HR radial	−0.414	0.325	0.106	−1.052	0.224
	**PWV**	0.964	0.341	0.116	0.296	1.632
	**SP carotid**	0.785	0.334	0.112	0.129	1.440
	**PP carotid**	0.942	0.340	0.116	0.275	1.608
	Eject duration carotid	0.563	0.328	0.108	−0.081	1.202
	**SEVR carotid**	−0.679	0.331	0.110	−1.329	−0.036
	**HR carotid**	0.656	0.331	0.109	0.008	1.305
	SP radial	−0.326	0.324	0.105	−0.961	0.309

BMI: body mass index; HOMA: Homeostatic Model Assessment for Insulin Resistance; AAT: abdominal adipose tissue; BMR: basal metabolic rate; PhA: phase angle; ECW/BCM: extracellular water to body cell mass ratio; DHEAS: dehydroepiandrosterone sulfate; D4-andro: Delta4-androstenedione; free-T: Free-testosterone; QT: the time from the start of the Q wave to the end of the T wave, time taken for ventricular depolarisation and repolarisation QTc: The corrected QT interval (QTc) estimates the QT interval at a standard heart rate of 60 bpm; QRS: The QRS complex is the combination of three of the graphical deflections seen on a typical electrocardiogram; T: The T wave is the positive deflection after each QRS complex; QRSD: the conduction through the specialized cardiac conduction system and ventricular myocardium; BRS: baroreflex sensitivity; mNN: mean N-N (or R-R) interval; SDNN: standard deviation of NN intervals; pNN50: The percentage of number of pairs of NN interval that is longer than 50 msec; SDANN: Standard Deviation of the 5 min Average NN intervals; ASDNN: the average of the SDNN of each 5-min segment over 24 h; rMSSD: Root mean square of the successive difference of NN interval; BB50: count of intervals that are more than 50 ms different from the previous interval; VLF: very low frequencies; IMT-L: intima media thickness left; IMT-R: intima media thickness right; PP: pulse pressure; SEVR: subendocardial viability ratio; HR: heart rate; PWV: pulse wave velocity; SP: systolic pressure.

**Table 2:** Significant correlations in the polycystic ovary syndrome (PCOS) group are presented in Hedge’s g and confidence intervals.

Markers	Hedge’s g [95% CI]
PhA/BMR	−0.829 [−1.543, −0.115]
PP/SP	−1.158 [−2.252, −0.063]

## Data Availability

The data cannot be publicly available due to GDPR privacy guidelines.

## Supplementary Materials

The following supporting information can be downloaded at: https://www.mdpi.com/article/10.3390/children9030316/s1, Table S1: Participants’ characteristics and measurements in mean (standard deviation SD); Table S2: Age correlations with adolescents’ significantly altered measurements in Spearman and *p*-value.

## Abbreviations

BMI: body mass index; HOMA: Homeostatic Model Assessment for Insulin Resistance; AAT: abdominal adipose tissue; PhA: phase angle; ECW/BCM: extracellular water to body cell mass ratio; DHEAS: dehydroepiandrosterone sulfate; D4andro: 4-androstenedione; free-T: Free-testosterone; QT: the time from the start of the Q wave to the end of the T wave, time taken for ventricular depolarisation and repolarisation QTc: The corrected QT interval (QTc) estimates the QT interval at a standard heart rate of 60 bpm; QRS: The QRS complex is the combination of three of the graphical deflections seen on a typical electrocardiogram; T: The T wave is the positive deflection after each QRS complex; QRSD: the conduction through the specialized cardiac conduction system and ventricular myocardium; BRS: baroreflex sensitivity; mNN: mean N-N (or R-R) interval; SDNN: standard deviation of NN intervals; pNN50: The percentage of number of pairs of NN interval that is longer than 50 msec; SDANN: Standard Deviation of the 5 min Average NN intervals; ASDNN: the average of the SDNN of each 5-min segment over 24 h; rMSSD: Root mean square of the successive difference of NN interval; BB50: count of intervals that are more than 50 ms different from the previous interval; VLF: very low frequencies; IMT-L: intima media thickness left; IMT-R: intima media thickness right; PP: pulse pressure; SEVR: subendocardial viability ratio; HR: heart rate; PWV: pulse wave velocity; SP: systolic pressure.
