# Supplementary material for: Hypertension Predisposition and Thermoregulation Delays in Adolescents with Polycystic Ovary Syndrome: A Pilot Study
Source: Children (Basel). 2022 Feb 25;9(3):316. doi: 10.3390/children9030316 (PMC8946960; doi:10.3390/children9030316)
Supplement: Supplementary file 1 [file children-09-00316-s001.zip › children-1548675-supplementary.pdf]

Table S1. Participants characteristics and measurements in mean(standard deviation SD)

FUNCTION	MARKER	PCOS mean(SD)	HEALTHY mean(SD)
Body Composition & Metabolism	AGE (years)	16.8(3.2)	16.9(2.1)
	BMI (kg/m²)	26.18 (4.04)	26.90 (5.89)
	AAT (cm²)	383.38 (193.80)	379.82 (169.71)
	BMR (kcal/day)	1327.41 (125.48)	1407.25 (128.8)
	PhA(°. degrees)	5.1 (1.30)	4.9(1.8)
	ECW/BCM	1.1 (0.24)	1.92(0.20)
Endocrine	Glucose mg/dL	91.87(7.94)	89.333(7.033)
	Insulin mIU/L	14.42	16.85
	free testosterone pg/mL	2.31(1.42)	2.56(1.27)
	DHEAS μg/dL	175.54(48.02)	232(142.83)
	D4 androstenedione ng/dL	22.67(9.7)	32.424(1.27)
Neuronal	BRS	2124(	23609(
	mNN	647.40 (203.69)	772.01(105.37)
	SDNN	124.27 (244.81)	70.26 (38.16)
	SDANN	22.40 (38.40)	16.20 (12.78)
	ASDNN	56.74 (11.81)	67.66 (33.69)
	rMSSD	32.94 (12.39)	40.00 (18.31)
	pNN50	26.93 (52.89)	20.43 (15.65)
	BB50	115.39 (98.90)	260.36 (236.88)
	VLF	383.45 (365.42)	954.04 (3701.2)
Cardiovascular	IMT-R	0.4505 (0.066)	0.430 (0.114)
	IMT-L	0.454 (0.055)	0.432 (0.119)
	SP radial	102.11(25.29)	110.33 (15.8)
	PP radial	30.56(8.68)	39.73(11.34)
	Eject duration radial	38.56(17.29)	41.8(9.001)
	SEVR radial	110.06 (44.91)	119.56 (38.31)
	HR radial	86.62(25.70)	86.8(19.84)
	***PWV**	52.87(5.87)	40.917(18.053)
	SP** ***carotid	119.79(10.30)	110.00(12.45)
	PP** ***carotid	48.929(10.118)	40.13(12.47)
	Eject duration *carotid*	39.5(7.002)	38.43(9.33)
	SEVR carotid	124.00(45.94)	146.71(40.302)
	HR carotid	86.71(16.77)	82.07(15.01)
	QT	332.5(108.601	371.187(24.089)
	QTc	368.667(121.821)	403.562(18.095)
	P	45.278(33.105)	33.125(30.908)
	QRS	57.722(29.132)	48.750(39.310)
	T	43.278(28.980)	30.71(30.646)
	QRSD	220.111(427.704)	87.14(9.984)
	HR	86.167(2.34)	86.5(9.817)

BMI: body mass index; AAT: abdominal adipose tissue; BMR: basal metabolic rate; PhA: phase angle; ECW/BCM: extracellular water to body cell mass ratio; DHEAS: dehydroepiandrosterone sulfate; D4andro: 4-androstenedione; free-T: Free-testosterone; QT:

the time from the start of the Q wave to the end of the T wave, time taken for ventricular depolarisation and repolarisation QTc: The corrected QT interval (*QTc*) estimates the QT interval at a standard heart rate of 60 bpm; QRS: The QRS complex is the combination of three of the graphical deflections seen on a typical electrocardiogram; T: The T wave is the positive deflection after each QRS complex; QRSD: the conduction through the specialized cardiac conduction system and ventricular myocardium; BRS: baroreflex sensitivity; mNN: mean N-N (or R-R) interval; SDNN: standard deviation of NN intervals; pNN50: The percentage of number of pairs of NN interval that is longer than 50 msec; SDANN: Standard Deviation of the 5 minute Average NN intervals; ASDNN: the average of the SDNN of each 5-minute segment over 24 hours; rMSSD: Root mean square of the successive difference of NN interval; BB50: count of intervals that are more than 50 ms different from the previous interval; VLF: very low frequencies; IMT-L: intima media thickness left; IMT-R: intima media thickness right; PP: pulse pressure; SEVR: subendocardial viability ratio; HR: heart rate; PWV: pulse wave velocity; SP: systolic pressure

Table S2. Age correlations with adolescents' significantly altered measurements in Spearman and p-value

**Markers**	**PCOS** p-value	**Controls** p-value
VLF/age	$p<0.05$	$p<0.05$
BMR/age	$p<0.05$	$p<0.05$
PhA/age	$p<0.05$	$p<0.05$
PWV/age	$p<0.05$	$p<0.05$
SEVR/age	$p<0.05$	$p<0.05$
PP/age	$p<0.05$	$p<0.05$
SP/age	$p<0.05$	$p<0.05$
Free testosterone/age	$p<0.05$	$p<0.05$
D4-androstenedione/age	$p<0.05$	$p<0.05$
